# Mutation-independent gene knock-in therapy targeting 5′UTR for autosomal dominant retinitis pigmentosa

**DOI:** 10.1038/s41392-022-01308-0

**Published:** 2023-03-08

**Authors:** Duc Anh Hoang, Baoshan Liao, Zongli Zheng, Wenjun Xiong

**Affiliations:** 1 Department of Biomedical Sciences, City University of Hong Kong, Hong Kong, China; 2 Key Laboratory of Biochip Technology, Biotech and Health Centre, Shenzhen Research Institute of City University of Hong Kong, Shenzhen, China; 3 TUNG Biomedical Sciences Centre, City University of Hong Kong, Hong Kong, China; 4 Ming Wai Lau Centre for Reparative Medicine, Karolinska Institutet, Hong Kong, China

**Keywords:** Gene therapy, Preclinical research

**Dear Editor**,

Retinitis pigmentosa (RP) is an inherited photoreceptor degeneration disease with high genetic heterogeneity (>90 disease-causing genes according to RetNet: https://web.sph.uth.edu/RetNet/sum-dis.htm). Taking a single RP disease gene *RHO* as an example, there are more than two hundred loss-of-function and gain-of-function mutations identified.^1^ While gene supplementation therapy has emerged as the most promising treatment for autosomal recessive RP (arRP) and X-linked RP (ClinicalTrials identifier: NCT01482195, NCT03328130, NCT03116113, NCT03252847, NCT03316560), therapeutic approaches to treat autosomal dominant RP (adRP) fall behind due to the low efficiency to disrupt mutant alleles specifically and a broad spectrum of the gain-of-function mutations.

The Rhodopsin protein, encoded by *RHO*, is the light-sensing G protein-coupled receptor that activates phototransduction in the rod photoreceptors. *RHO* mutations are responsible for 20–30% of adRP, with the *Rho*^*P23H*^ (p.Pro23His, c.68 C > A) mutation being the most common mutation in adRP patients.^2,3^ Here we present a mutation-independent gene editing strategy to treat *RHO*-associated adRP. Briefly, AAV-Cas9-mediated gene knock-in (KI) was targeted to the 5′ untranslated region (UTR) of the *Rho* gene by homology-independent targeted integration (HITI) (Fig. 1a).^4^ Two gRNAs of SpCas9 that are immediately upstream of the Kozak sequence of mouse *Rho* gene were chosen (Fig. 1b). The gRNA (gRNA1) with higher cutting efficiency (Supplementary Fig. 1) was used for in vivo tests. To ensure the fitting of SpCas9 in the AAV vector, a short hRK promoter is used to drive SpCas9 expression in the photoreceptor cells specifically (Fig. 1c). The two AAV vectors, one packaging hRK-Cas9 and the other packaging hRK-mCherry, pU6-gRNA1 and the HITI donor sequence, were subretinally injected into neonatal mouse eyes (Fig. 1c). In the event of HITI AAV-mediated GFP KI, GFP was specifically expressed in the transduced rods but not in the cones (Fig. 1d, Supplementary Fig. 2). Rho KI was confirmed by RHO staining in the *Rho*^*−/−*^ mice,^5^ which retain the gRNA1 target site in *Rho* 5′UTR (Fig. 1d, Supplementary Fig. 3). To quantify the AAV transduction and GFP KI efficiencies, we counted the mCherry + , GFP + and DAPI + cells in the outer nuclear layer of the retinal sections and showed that the AAV transduction efficiency is 46.2% of all photoreceptors and the GFP KI efficiency is 48.1% of the transduced photoreceptors and 21.9% of all photoreceptors (Fig. 1e). Next generation sequencing (NGS) using purified transduced photoreceptors showed that the in vivo KI efficiency was as high as 43% (close to the quantification on the retinal sections), INDEL rate 44% and unmodified alleles 13% (Fig. 1f). Together we show that the HITI-AAV approach can mediate efficient gene KI into *Rho* 5′UTR.

**Fig. 1:**
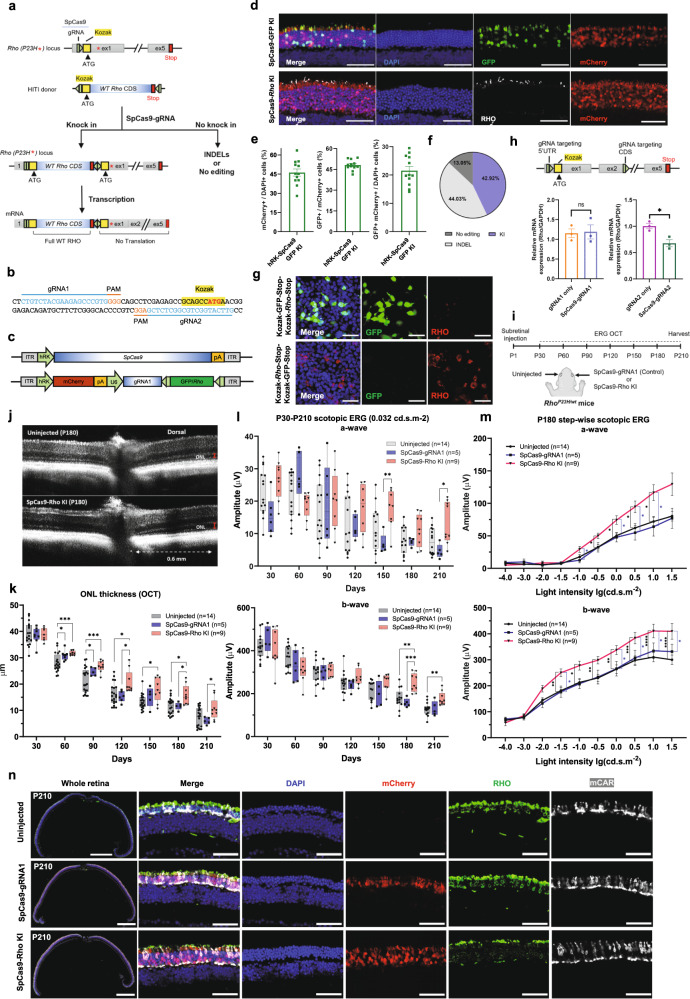
Rescuing visual function of the *Rho*^*P23H/wt*^ mice via 5′UTR rhodopsin gene integration mediated by CRISPR/Cas9. **a** Schematic representation of gene KI into 5′UTR *Rho* locus based on the HITI method. Green pentagon, SpCas9-gRNA targeted region; Black line within the pentagon, the cleaved site; Gray rectangle, exon; Yellow rectangle, Kozak sequence; Red rectangle, stop codon sequence; Red asterisk, P23H mutation; HITI donor, the *Rho* CDS flanked by two gRNA targeting sequences. **b** SpCas9-gRNA targeted sequences. Sequence in blue, the SpCas9-gRNAs targeting regions; Sequence in orange, PAM sequence; Region highlighted by yellow, Kozak sequence; Bold sequence labeled by red, ATG start codon. **c** Schematic of dual AAV vectors delivering SpCas9, mCherry, gRNA1, and GFP- or *Rho*-HITI donor into mouse retina. Both SpCas9 and mCherry reporter were driven by the hRK promotor. gRNA1 expression was driven by the U6 promoter. **d** Representative retinal sections of *Rho*^*−/−*^ mice received GFP and *Rho* KI mediated by AAV8-SpCas9 and AAV8-gRNA1-GFP/*Rho* HITI donor. Scale bar, 50 μm. **e** Quantifications of AAV transduction efficiency (mCherry+/ DAPI + %), KI efficiency of the transduced cells (GFP+/mCherry+%), and KI efficiency of all photoreceptors (GFP + mCherry+/DAPI + %) in the retinal sections treated with GFP KI AAV vectors (*n* = 12). Only cells in the outer nuclear layer were counted. **f** NGS results showing allele frequencies of *Rho* knock-in (KI), INDEL, and no editing in the purified mCherry+ photoreceptor cells. **g** Representative images of 293 T cells transfected by Kozak-GFP-Stop-Kozak-*Rho*-Stop or Kozak-*Rho*-Stop-Kozak-GFP-Stop plasmid and stained for RHO. Scale bar, 50 μm. **h** qPCR results showing the *Rho* expression levels in the wild-type retinas with *Rho* 5′UTR or CDS INDELs by two different gRNAs. *n* = 3 retinas for all groups. A schematic representation of genome editing sites by the two gRNAs respectively was shown on the top. Green pentagon, gRNAs targeting 5′UTR or CDS of *Rho*; Black line within the pentagon, the cleaved site; Gray rectangle, exon; Yellow rectangle, Kozak sequence; Red rectangle, stop codon. **i** Experimental design of therapeutic efficacy test in the *Rho*^*P23H/wt*^ mice. Eyes were untreated, treated with AAV8-SpCas9 + AAV8-mCherry-U6-gRNA1 (labeled as SpCas9-gRNA1), or treated with AAV8-SpCas9 + AAV8-mCherry-U6-gRNA1-*Rho* donor (labeled as SpCas9-Rho KI). **j** Representative OCT images of P180 *Rho*^*P23H/wt*^ retinas that were untreated or treated with SpCas9-Rho KI. **k** ONL thickness of *Rho*^*P23H/wt*^ retinas with different treatments from P30 to P210. **l** P30-P210 a- and b-wave amplitudes of rod scotopic ERG responses of control and treated *Rho*^*P23H/wt*^ eyes under light intensity 0.032 cd.s.m^−2^. **m** a- and b-wave amplitudes of step-wise scotopic ERG responses of P180 *Rho*^*P23H/wt*^ mice eyes by light intensity from −4.0 lg(cd.s.m^−2^) to 1.5 lg(cd.s.m^−2^). Black asterisks indicate significant differences between the untreated retinas and the retinas treated with SpCas9-Rho KI; blue asterisks indicate significant differences between the retinas treated with SpCas9-gRNA1 and SpCas9-Rho KI. **n** Representative retinal sections of *Rho*^*P23H/wt*^ mice at P210. Sections were immunostained with anti-mCAR (cone marker, in white) and anti-RHO (rod marker, in green) antibodies. Scale bar in the left-most column: 500 μm. Scale bar in the other columns: 50 μm. Data are presented as mean ± s.e.m. ns. not significant, *P < 0.05; **P < 0.01; ***P < 0.001; ****P < 0.0001, by unpaired two-tailed Student t-test (h) and two-way ANOVA with Tukey post hoc test (k-m)

Two potential circumstances may prevent 5′UTR gene insertion from working properly. The first one is that the endogenous *Rho*^*P23H*^ allele, which retains the intact Kozak sequence, may continue to express the toxic protein. To address this concern, we tested whether the expression of the downstream gene is completely blocked by the inserted sequence. A dual Kozak reporter construct was made by cloning the expression cassette (the *Rho* CDS with 5′UTR GFP insertion) into a pCMV vector, and another construct was made with the reversed order of *Rho* and GFP CDS (Supplementary Fig. 4). The transfection result of both constructs demonstrated that only the gene placed upstream was expressed while the protein product of the downstream gene was undetectable (Fig. 1g). Second, as INDELs near the translation starting site might lead to a lower level of *Rho* expression and thereby affect visual function, we examined the impacts of INDELs in the *Rho* locus on *Rho* expression level and mouse visual function. C57BL/6 mice were injected with dual vectors without HITI donor to create INDELs. mCherry+ photoreceptors were isolated for *Rho* mRNA level examination. The qPCR result showed that *Rho* mRNA was not significantly affected by 5′UTR INDELs, in contrast to a significant expression decrease by CDS INDELs (Fig. 1h). Visual functions tested by the optomotor assay and electroretinography (ERG) showed that the visual acuity and light-induced potential change were not affected in the 5′UTR-targeted group (Supplementary Fig. 5). Thus, we conclude that INDELs in 5′UTR do not affect endogenous *Rho* expression or visual function.

To test the therapeutic effect of 5′UTR Rho KI, we treated the *Rho*^*P23H/wt*^ mice, which have progressive photoreceptor degeneration as human patients with the same mutation,^5^ with the dual AAV vectors on P1 (Fig. 1i). Monthly retina structure examination by optical coherence tomography (OCT) revealed significant rescue of photoreceptor layer thickness from P60 to P210 in the Rho KI group in comparison to the control groups (Fig. 1j, k). The ERG results showed that Rho KI eyes have significantly higher scotopic ERG a- and b-wave amplitudes under dim light conditions at late degeneration stages, suggesting the better light-sensing function of the rods (Fig. 1l, m). Both mixed rod-cone scotopic ERG and cone-dominant photopic ERG showed increased amplitude with Rho KI from P180 to P210 (Fig. 1m, Supplementary Fig. 6), suggesting a rescue of the secondary cone degeneration. Histological analysis of the P210 harvested eyes showed that Rho KI treatment preserved a thicker photoreceptor cell layer with more rods (Fig. 1n, Supplementary Fig. 7), and the remaining rods had better morphology with longer inner/outer segments (Fig. 1n, Supplementary Fig. 7). A set of animals were aged to P480 for the long-term follow-up study. At P480, the RHO staining was largely undetectable in the untreated and control-treated retinas, while it was still present across the whole retinal section of the Rho KI treated group, suggesting that the rescue effect is long-lasting (Supplementary Fig. 8). In all assays, the control treatment without HITI *Rho* donor did not provide any beneficial effect or induce any toxic effect in *Rho*^*P23H/wt*^ mice (Fig. 1j–n). Together, these results showed that AAV-mediated Rho KI into the 5′UTR in an allele-independent manner efficiently hampers rod degeneration and vision loss in *Rho*^*P23H/wt*^ mice.

Recently AAV-HITI-mediated gene KI targeting the disease gene CDS was shown in both mouse retina and liver, demonstrating the feasibility of gene KI therapy for treating dominant diseases.^5^ Here we propose that gene KI targeting 5′UTR offers good efficacy and safety. First, the inserted sequence, which contains the translation initiation element, does not need to be in frame with the endogenous CDS. Second, the STOP codon of the inserted gene in the 5′UTR will block the expression of the downstream endogenous gene, while CDS KI may lead to the expression of truncated proteins which may function dominant-negatively. Moreover, 5′UTR INDELs do not abolish endogenous gene expression in the cells without successful KI, while CDS INDELs may create a reading frame shift and a knockout effect of the wild-type allele. PAM sites of various Cas9 enzymes are readily available and conserved in the upstream sequence of Kozak in the 5′UTR of the most common adRP disease genes (Supplementary Fig. 9), suggesting that 5′UTR gene KI may be widely applicable. Altogether, we have developed a mutation-independent gene KI approach that targets 5′UTR of both alleles of the disease gene and demonstrated its therapeutic potential in an animal model of mutant allele-dominant disease.

## Supplementary information

Supplementary materials

## Data Availability

All data and materials shown in the main manuscript or supplementary materials can be obtained from the corresponding authors as reasonably required.
