# Supplementary material for: Mutation-independent gene knock-in therapy targeting 5′UTR for autosomal dominant retinitis pigmentosa
Source: Signal Transduct Target Ther. 2023 Mar 8;8:100. doi: 10.1038/s41392-022-01308-0 (PMC9992370; doi:10.1038/s41392-022-01308-0)
Supplement: Supplementary file 1 — Supplementary materials [file 41392_2022_1308_MOESM1_ESM.docx]

**Supplemental Materials for**

**Mutation-independent gene knock-in therapy targeting 5’UTR for autosomal dominant retinitis pigmentosa**

Duc Anh Hoang^#1,2,3^, Baoshan Liao^#1,2,3^, Zongli Zheng^1,4^, Wenjun Xiong^1,2,3^

1. Department of Biomedical Sciences, City University of Hong Kong, Hong Kong, China.

2. Key Laboratory of Biochip Technology, Biotech and Health Centre, Shenzhen Research Institute of City University of Hong Kong, Shenzhen, China.

3. TUNG Biomedical Sciences Centre, City University of Hong Kong, Hong Kong, China.

4. Ming Wai Lau Centre for Reparative Medicine, Karolinska Institutet, Hong Kong, China.

# These authors contributed equally to this work.

***Corresponding author:** Wenjun Xiong, Ph.D.,

Department of Biomedical Sciences, City University of Hong Kong, Tat Chee

Avenue, Kowloon, Hong Kong, China.

Tel: 852-3442 2494

Fax: 852-3442 0549

Email:  wenjun.xiong@cityu.edu.hk

**This file includes:**

Materials and Methods

Supplementary figures 1 to 9

References

**Materials and Methods**

**Animals**

The *Rho^P23H/wt^* and C57BL/6J mice were purchased from the Jackson Laboratory. The *Rho*^-/-^ mouse was obtained from Janis Lem (Tufts University, Boston, Massachusetts, USA)^1^. The *Rho^P23H/wt^* and *Rho*^-/-^ mice were genotyped by PCR method with the primer set suggested by the original publications^1,2^. All mice were kept on a 12h light/12h dark cycle in the Laboratory Animal Research Unit, City University of Hong Kong. All animal procedures performed were approved by the Hong Kong Department of Health under Animals Ordinance Chapter 340 (Ref: (20-130) in DH/HT&A/8/2/5 Pt.2) and by the City University of Hong Kong Animal ethics committee (Ref: A-0264).

**Plasmid construction**

The plasmid for knock-out efficiency testing: pAAV-CMV-SpCas9-2A-mCherry-bGHPA-U6-gRNA for SpCas9 gRNAs knock-out efficiency testing was constructed as previously described^3^. In brief, the SaCas9 cassette of pAAV-CMV-SaCas9-U6-Bsal-gRNA (Addgene no.61591) was replaced by SpCas9, which was amplified from LentiV-Cas9-puro (Addgene no.108100), via AgeI and BamHI site. gRNA1 and gRNA2 (primer sets Rho gRNA1/2 Sp F and R) were inserted into pAAV-CMV-SpCas9-2A-mCherry-bGHPA-U6-gRNA via BsaI site.

The AAV virus plasmids: For testing the gene knock-in therapy, pAAV-hRK-SpCas9, pAAV-hRK-mCherry-U6-gRNA1, pAAV-hRK-mCherry-U6-gRNA1-*Rho*-HITI donor, and pAAV-hRK-mCherry-U6-gRNA1-GFP-HITI donor were constructed. pAAV-hRK-SpCas9 was made by replacing the IZsGreen in pAAV-hRK- IZsGreen (gift from the T. Li Laboratory, National Eye Institute, Bethesda, MD^4^) with the SpCas9 sequence. pAAV-hRK-mCherry-U6-gRNA1 was cloned by inserting hRK-mCherry via Xbal&BamHI first and inserting gRNA1 sequence via BsaI site next into the backbone plasmid pAAV-CMV-SaCas9-U6-Bsal-gRNA (Addgene no.61591). To construct the *Rho*-HITI donor, *Rho* CDS was amplified from the retinal cDNA of C57BL/6J with the primers containing CRISPR/Cas9 gRNA1 targeted site (*Rho* CDS F and R) and inserted into the backbone vector pAAV-hRK-mCherry-pA-U6-gRNA1 to generate pAAV-hRK-mCherry-pA-U6-gRNA1-Rho HITI donor. pAAV-hRK-mCherry-pA-U6-gRNA-GFP HITI donor was constructed in a similar method.

Dual Kozak reporter plasmids with KI sequence: The plasmids containing the fragment of HITI donor integrated into *Rho* locus, pCMV-Kozak-GFP-Stop-Kozak-*Rho*-Stop and pCMV-Kozak-*Rho*-Stop-Kozak-GFP-Stop, were constructed. For the pCMV-Kozak-GFP-Stop-Kozak-*Rho*-Stop, the Kozak-*Rho-*Stop was amplified and inserted into pCMV backbone plasmid via restricted enzyme sites (SalI and MluI) to create the pCMV-Kozak-*Rho-Stop*. Next, the Kozak-GFP-Stop fragment, mimicking the integrated fragment of 5’-UTR *Rho* induced by SpCas9-*Rho* KI, was amplified and sub-cloned into the pCMV-Kozak-*Rho-*Stop to generate the pCMV-Kozak-GFP-Stop-Kozak-*Rho*-Stop. A similar approach was used to construct the pCMV-Kozak-*Rho*-Stop-Kozak-GFP-Stop.

**Primer list**

| Name | Sequence (5’–3’) | Purpose |
| --- | --- | --- |
| *Rho* gRNA1-Sp F | CACCGCTGTCTACGAAGAGCCCGTG | Construct gRNA1 for SpCas9 targeting *Rho* ATG upstream |
| *Rho* gRNA1-Sp R | AAACCACGGGCTCTTCGTAGACAGC |  |
| *Rho* gRNA2-Sp F | CACCCGTTCATGGCTGCGGCTCTCG | Construct gRNA2 for SpCas9 targeting *Rho* ATG upstream |
| *Rho* gRNA2-Sp R | AAACCGAGAGCCGCAGCCATGAACC |  |
| *mRho* exon 1 F | TGATATCTCGCGGATGCTGAAT | *Rho^P23H/wt^* mouse genotyping |
| *mRho* exon 1 R | TGGGCCTTTAGATGAGACCAA |  |
| *Rho*_Neo F | CGGGAGCGGCGATACCGTAAAGC | *Rho^-/-^* mouse genotyping |
| *Rho*_Neo R | GAAGCGGGAAGGGACTGGCTGCTA |  |
| *Rho* CDS F | AAACGCGACGCGTCCCCACGGGCTCTTCGTAGACAGAGCCGCAGCCATGAACGGCACAGAGGGCCCCAATTTTTATG | *Rho*-HITI donor construction |
| *Rho* CDS R | CCAGGTGGCTCCAGCCTAATAAGTCGACCCCCACGGGCTCTTCGTAGACAGGC |  |
| AgeI GFP F: | GTGAACACCGGTAGACAGAGCCGCAGCCATGGTGAGCAAGGGCGAGG | Construct pCMV-Kozak-GFP-Stop-Kozak-*Rho*-Stop |
| SalI GFP R: | TTGGCGCGCCGTCGACTTATTACTTGTACAGCTCGTCCATGC |  |
| Kz-*Rho* CDS-stop F | GAGGTCTATATAAGCAGAGCTCTCTGGCTAACTAAGCCGCAGCCATGAACGGCACAGAG | Construct pCMV-Kozak-*Rho*-Stop-Kozak-GFP-Stop |
| Kz-*Rho* CDS-stop R | GCGGCTCTGTCTACGAAGAGCCCGTGGGGACGCGTTAGGCTGGAGCCACCTGGCTGGTC |  |
| *Rho* KI_F | GCTGAGCTCGCCAAGCAGCCTTGGT | *Rho* KI NGS sequencing |
| *Rho* KI_R | CATGTACGCTGCCAGCATGGAGAAC |  |

**Analysis of CRISPR/gRNA editing efficiency in MEF cells**

MEF cells were cultured in DMEM, 10% (v/v) FBS, 1% (v/v) Penicillin/Streptomycin (P/S) media in 37℃, 5% CO_2_ incubator. Plasmids were transfected into MEF cells using lipofectamine 3000 (Thermo Fisher Scientific). The lipofectamine 3000 was mixed with plasmid in ratio 3:1 in DMEM medium (200ul/1ug plasmid) and incubated for 10-15 mins at room temperature. The mixture was subsequently added into MEF cells with DMEM, 2% (v/v) FBS, 1% (v/v) Penicillin/Streptomycin medium and placed in 37℃, 5% CO_2_ incubator. The old medium was removed and replaced with the fresh DMEM 24 hours after transfection. The transfected MEF cells with mCherry marker were sorted by FACS three days after transfection. Genomic DNA was amplified using the *mRho* exon 1 F and R primers listed in the primer table. PCR products were sequenced by the Sanger sequencing performed by BGI Genomics. The sequencing results were subsequently uploaded to ICE CRISPR Analysis (https://ice.synthego.com) for analyzing the editing efficiency.

**AAV packaging and titration**

Recombinant AAV8 vectors were produced in HEK293T cells, and the AAV purification process is based on the iodixanol gradient method^5^. The HEK293T cells were transfected at 80-90% confluency. For 5 plates (150mm) of HEK293T cells, 35ug of pAAV vector transgene plasmid, 35ug of Rep/Cap 2/8 packaging plasmid, and 100 ug adenoviral helper plasmid pAdDeltaF6 (Rep/Cap 2/8 and pAdDeltaF6 obtained from the Penn Vector Core, University of Pennsylvania) were mixed with 510ug of polyethyleneimine (PEI) (DNA:PEI=1:3) in DMEM. The mixture was incubated for 15 mins at room temperature before adding to the HEK293T cells cultured in DMEM, 10% (v/v) Nu-Serum (355500, Corning), 1% (v/v) P/S. After 24 hours, the old medium was changed with fresh DMEM, 1% (v/v) P/S. 72 hours after transfection, the AAV8 supernatant was collected and centrifuged 2000g, 15 mins, 4℃, to remove cell debris. After cell debris clarification, the supernatant was mixed with 8.5% (w/v) PEG-8000, 0.4 M NaCl at 4℃ for 2 hours, to precipitate the AAV virus. The precipitation particles were then collected by centrifugation at 7,000 × g for 10 mins, 4℃, and resuspended in the virus lysis buffer (150 mM NaCl and 20 mM Tris, pH 8.0). The virus mixture was further purified by ultra-centrifugation in the iodixanol step gradient at 147,000 x g at 4℃ for 90 mins. The AAV residing at 40% iodixanol fraction was collected and washed three times with PBS by using Amicon 100K columns (EMD Millipore). About 200µl of final volume AAV was collected and stored at -80℃. Virus titration was performed by the protein SDS-PAGE method.

**Subretinal injection of AAV**

AAV8-hRK-Cas9 and AAV8-hRK-mCherry-pU6-gRNA-donor viruses were mixed in PBS to a final concentration of 5E12 vg/ml for each virus. New-born mouse pups were anesthetized by hypothermia on ice for 2-3 mins. An incision was made in the eyelid to expose the eyeball. 0.25ul of virus mix was injected into the subretinal space using a pulled angled glass pipette controlled by a FemtoJet (Eppendorf). The right eye of the animal was injected, and the left eye was uninjected for control.

**Fluorescence-activated cell sorting (FACS) of the transduced photoreceptors**

The photoreceptors were transduced by dual AAV vectors pAAV-hRK-SpCas9 and pAAV-hRK-mCherry-U6-gRNA1-*Rho*-HITI in *Rho^P23H/wt^* neonatal mice as described above. 14 days after injection, retinas were dissected from the enucleated eyeballs and disassociated using papain solution. 200k mCherry-positive cells (transduced photoreceptors) were sorted by Sony SH800 Cell Sorter and collected for DNA extraction.

**DNA extraction**

Genomic DNA (gDNA) was extracted from the sorted transduced photoreceptors using the PureLink™ Genomic DNA Mini Kit (Thermo Fisher Scientific). Briefly, the cells were collected and rinsed two times with PBS. After rinsing, cells were treated with DNA lysis buffer containing protease K and RNAase at 55℃ for 1 hour or until cell pellets were dissolved. Next, the cell lysis mixture was thoroughly mixed with DNA binding buffer and ethanol before flowing through the binding column by centrifugation 10,000g for 1 min. After binding, the column was washed two times with 500µl of washing buffer. Finally, the gDNA was recovered in 50 ul EB buffer and kept at -20℃ for further analysis.

**NGS analysis**

A pair of primers (Rho KI_F and Rho KI_R) flanking the upstream and downstream of SpCas9-gRNA1 target site was designed to amplify the extracted gDNA of the purified transduced photoreceptors. Phusion High-Fidelity DNA Polymerase (New England Biolabs) was used for precise amplification. The PCR product (~200bp in size) was further isolated by gel electrophoresis and purified with TIANgel Midi Purification Kit (TIANGEN) for NGS. Paired-end 150bp reading was adopted to cover the whole length of PCR product using Illumina NovaSeq 6000 platform. 1 million reads were generated from NGS and were analyzed by CRISPResso for editing efficiency evaluation. To quantify the overall SpCas9 cleavage efficiency in mouse photoreceptor cells, we used CRISPResso2^6^ with a quantification window centered on the cutting position and a ±5bp window to count short INDEL and KI reads. Successful *Rho* CDS integration introduced a KI marker sequence, which consists of the gRNA target sequence and its reverse complement sequence (5’-GTCTACGAAGAGCCCGGGCTCTTCGTAGAC-3’). The HITI KI efficiency in mouse photoreceptor cells was calculated as the proportion of the reads with the KI marker sequence among the overall reads. The INDEL efficiency was calculated as the proportion of the reads with short INDELs among the overall reads. Sequencing data are released on Sequence Read Archive (SRA) database (<https://www.ncbi.nlm.nih.gov/sra/>) under the accession code PRJNA901526.

**Electroretinography (ERG)**

The eye physiology of mice was determined by ERG measurements using Espion E3 System (Diagnosys LLC). The ERG protocol was developed from the previous studies of wild-type mice to characterize the rod and cone responses^7,8^. The mice were adapted in a dark cabinet overnight before ERG testing, then were anesthetized with a ketamine/xylazine (100/10 mg/kg) mixture. The eyes were dilated with a drop of 5% phenylephrine (Mydrin-P, Santen Pharmaceutical Co) and 0.5% tropicamide solution for 5 mins and were kept hydrated with corneal gel. After putting mice on the platform, gold-wire electrodes for measuring electrical responses were placed on each eye cornea, while a reference electrode and a ground electrode were put into the mouth and the tail, respectively. All these steps were performed in the darkroom under dim red light. For scotopic ERG recordings, a multiple 530nm light with different intensities (increments from 0.01 cd s/m^2^ to 30 cd.s/m^2^) were elicited to stimulate scotopic responses in a specific time interval. For photopic ERG recordings, 5 mins exposure under 10 cd.s/m^2^ light intensity was adopted to inhibit the rod function. The photopic response was measured by multiple flashes of 30 cd.s/m^2^ intensity in the illuminated background (10 cd.s/m^2^). The average amplitude and implicit time of a- and b-wave were recorded and exported for further analysis.

**Optical Coherence Tomography (OCT)**

In vivo OCT was performed using a Bioptigen spectral domain optical coherence tomographer (SD-OCT, Bioptigen Envisu R4310 SD-OCT, Germany). Before the procedure, mice were anaesthetized by intraperitoneal injection with a standard mixture of ketamine/xylazine (100 mg ketamine + 10 mg xylazine)/ kg body weight. Mice were provided supplemental indirect warmth by a heating pad during anesthesia. Cornea and pupil were anaesthetized and dilated with instillation of 0.5% proxymetacaine hydrochloride (Provain-POS), and 0.5% tropicamide and 0.5% phenylephrine hydrochloride (Mydrin-P, Santen Pharmaceutical Co) solution. During retinal imaging, cornea was hydrated with lubricating eye drops (Systane Ultra, Alcon). To image the retina, the volume intensity projection was centered on the optic nerve, and the following scan parameters were applied: radial volume scans 1.7 mm in diameter, 1000 A-scans/B-scan, 8 B-scans/volume, 24 frame/B-scan, 80 inactive A-scans/B-scan, and 1 volume. The ONL thickness was measured at a distance of 0.6mm away from the optic nerve head using ImageJ.

**Retinal section and histology**

The mice were sacrificed by CO_2_ euthanasia and cervical dislocation. The eyeballs were dorsally marked before enucleation, and then retinas were dissected and fixed in 4% formaldehyde for 30 mins at room temperature. Fixed retinas were washed three times with PBS and sequentially cryoprotected in 5%, 15%, and 30% sucrose for 15, 30, and 60 mins, respectively. Next, the eyecups were soaked in optimal cutting temperature (OCT) and 30% sucrose solution 1:1 at 4℃ overnight and subsequently embedded in cryomold in orientation to achieve dorsal-ventral oriented slice or infected-uninfected oriented slice. After tissue freezing below -20℃, a series of 20μm sections were cut and collected in the glass slides using a cryostat machine (Thermo HM525NX Cryostat). In the immunostaining process, the retinal sections or whole retinal cups were firstly incubated in the blocking solution (3% BSA, 0.1% Triton X-100 in PBS - PBST) for 30 mins, then were covered with primary antibodies at recommended dilution at 4 °C, overnight. After primary antibody incubation, the samples were washed three times with PBST before incubation in a mixture of DAPI (0.5 μg/ml) and secondary antibodies in the dark for 2 hours at room temperature. The primary antibodies and secondary antibodies are listed in the following table. Before microscopy processing or cool storage, the sections or whole flat-mount retinas were mounted with an anti-fade solution. Slide images were captured in Zeiss LSM780 confocal microscope or Nikon Eclipse Ni-E upright microscope. The histology measurements and image processing were performed in ImageJ software.

List of antibodies used for immunostaining

| Name | Type | Host | Working dilution | Brand (Cat.) | Purpose |
| --- | --- | --- | --- | --- | --- |
| mCAR | 1^st^ antibody | Rabbit | IHC(1:500) | Millipore  (AB15282) | Label cone cells |
| RHO 4D2 | 1^st^ antibody | Mouse | IHC(1:500) | Millipore  (MABN15) | Label rod outer segments |
| Anti-Mouse | 2nd antibody  Alexa 488 | Donkey | IHC(1:1000) | Jackson ImmunoResearch  (715-545-150) | Label mouse antibody with green fluorescent |
| Anti- Mouse | 2nd antibody  Alexa 647 | Donkey | IHC(1:1000) | Jackson ImmunoResearch  (715-605-150) | Label mouse antibody with magenta fluorescent |
| Anti- Rabbit | 2nd antibody  Alexa 488 | Donkey | IHC(1:1000) | Jackson ImmunoResearch  (711-545-152) | Label rabbit antibody with green fluorescent |
| Anti-Rabbit | 2nd antibody  Alexa 647 | Donkey | IHC(1:1000) | Jackson ImmunoResearch  (711-605-152) | Label rabbit antibody with magenta fluorescent |

**Statistical Analysis**

Data were presented as mean ± s.e.m. Sample sizes were indicated for each experiment in the figure legend. One-way or two-way ANOVA analysis followed by the Tukey test was performed to compare multiple groups, and the unpaired two-tailed Student’s t-test was used to compare two groups.

**Supplementary Figures**

**
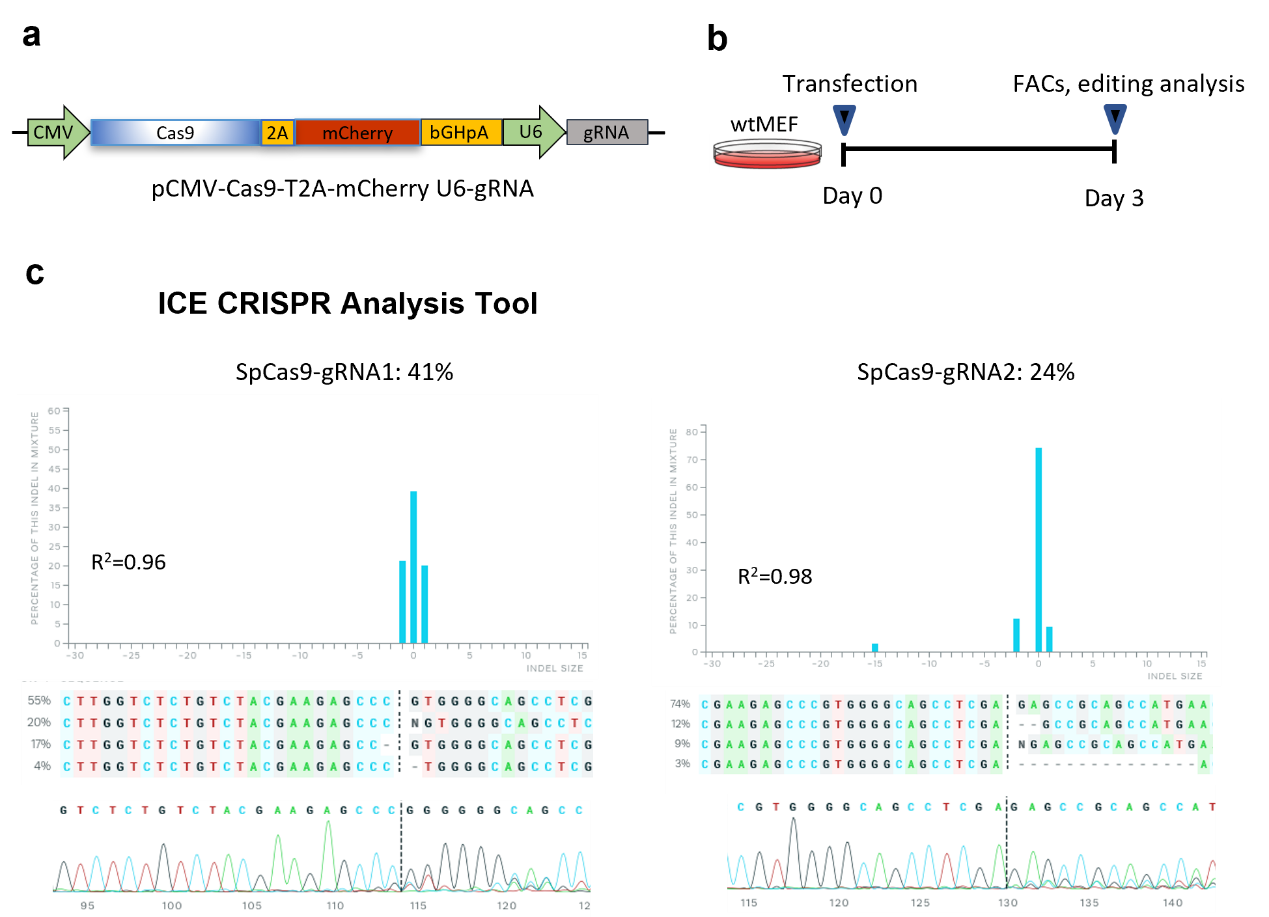
**

**Supplementary figure 1. In vitro screening of SpCas9 gRNAs targeting the *Rho* 5’UTR. a.** Schematic of the expression plasmid for screening Cas9 gRNAs targeting efficiency. CMV and U6 are promoters of driving transcription of Cas9, mCherry, and gRNA, respectively. BGHpA, Bovine Growth Hormone Polyadenylation. 2A, self-cleaving peptides sequence. **b.** Timeline of Cas9-gRNA targeting efficiency evaluation. The Cas9-gRNA plasmids were transfected into wild-type MEF cells, and the mCherry+ cells were sorted after 3 days of transfection for genomic DNA extraction and gene editing analysis. **c.** The SpCas9-gRNAs targeting efficiencies were analyzed by ICE CRISPR Analysis tools, and SpCas9-gRNA1 showed the higher knock-out efficiency, 41%.


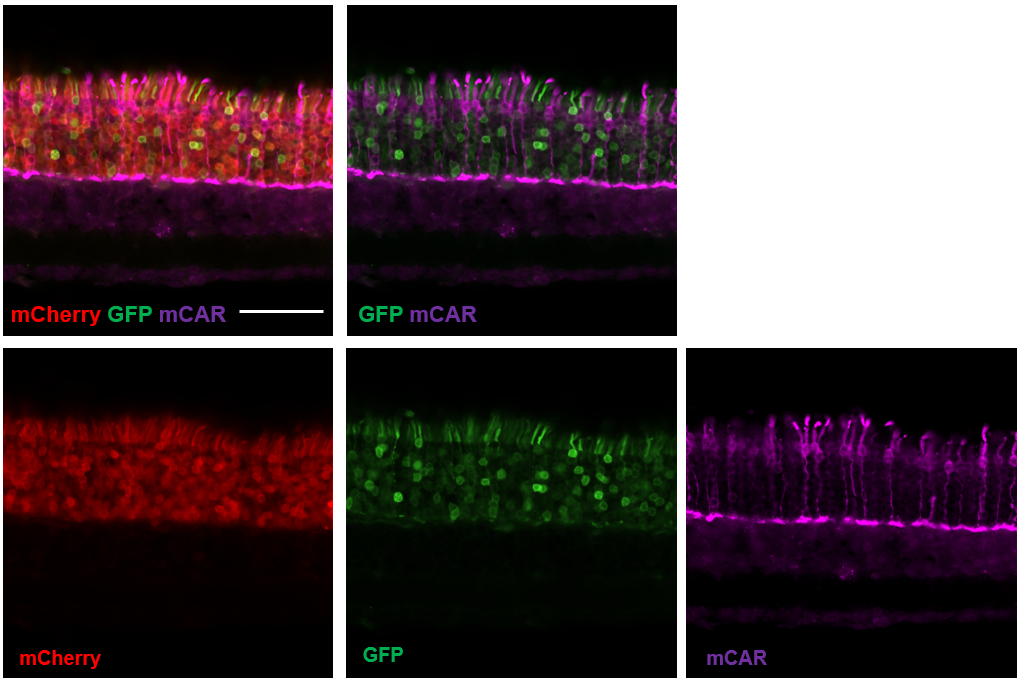


**Supplementary figure 2. Specific expression of the inserted GFP in the rods in the mouse retina that was treated with SpCas9-GFP KI.** Representative retinal section images of wild-type mice received SpCas9-GFP KI treatment. Retinal sections were immunostained with anti-mCAR (cone marker, in magenta) antibody and imaged for mCherry (red) and GFP (green) fluorescence. Scale bar, 50 μm.

**
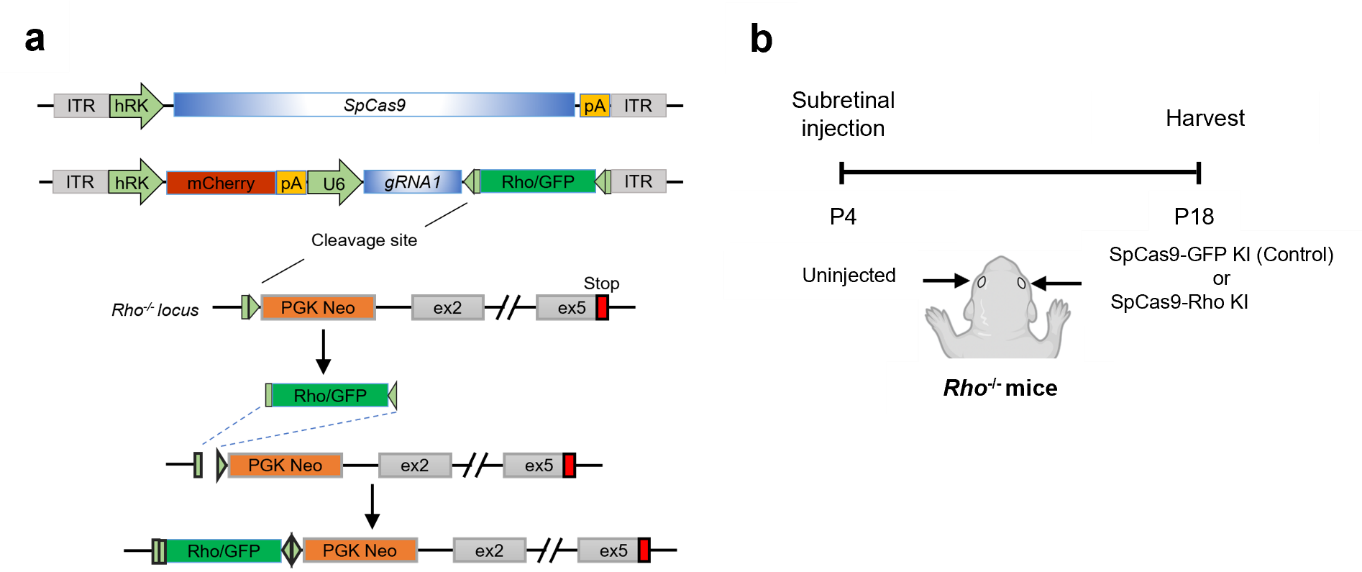
**

**Supplementary figure 3. Schematic diagram showing *Rho* or GFP integration in the *Rho^-/-^* mice.** **a.** Schematic of AAV vectors delivering SpCas9, gRNA, and *Rho* or GFP donor to the retina. hRK promoter controls the expression of SpCas9 and mCherry. gRNA1 was controlled by the U6 promoter. The donor was flanked by 2 targeting sites of SpCas9-gRNA1. In RHO KO transgenic mice, the *Rho* exon1 was replaced by PGK Neo cassette; However, the SpCas9-gRNA targeting site was not changed. **b.** Experimental design of CRISPR/Cas9 mediated *Rho* or GFP integration in *Rho^-/-^* mice.

**
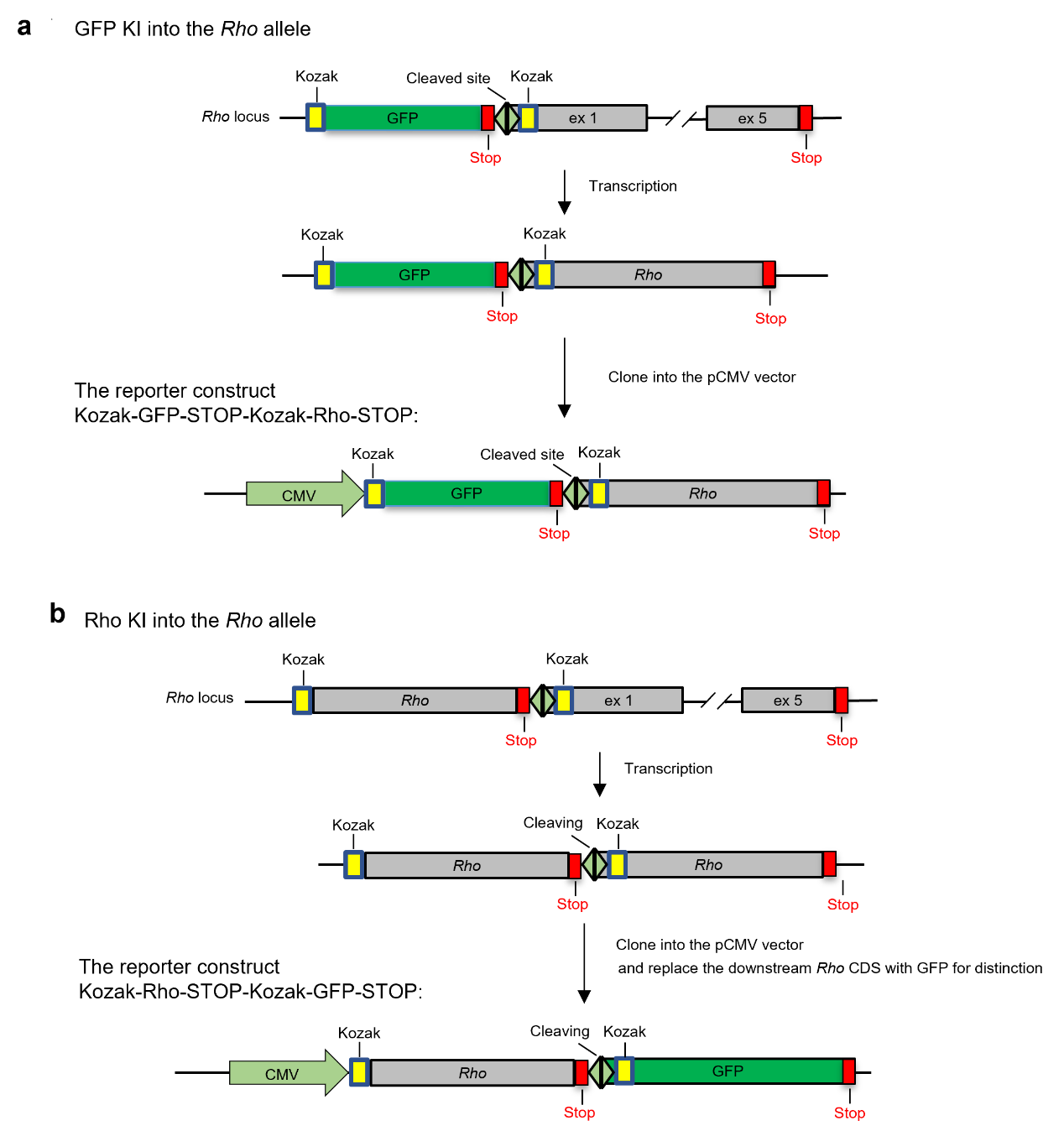
**

**Supplementary figure 4. Gene integration in the 5’UTR disrupts the original *Rho* expression.** **a.** Schematic representation of GFP knock-in into *Rho* locus mediated by SpCas9–gRNA1 and the reporter construct of CMV-Kozak-GFP-Stop-Kozak-*Rho*-Stop. **b.** Schematic representation of *Rho* CDS knock-in into *Rho* locus mediated by SpCas9–gRNA1 and the reporter construct of CMV-Kozak-*Rho*-Stop-Kozak-GFP-Stop.


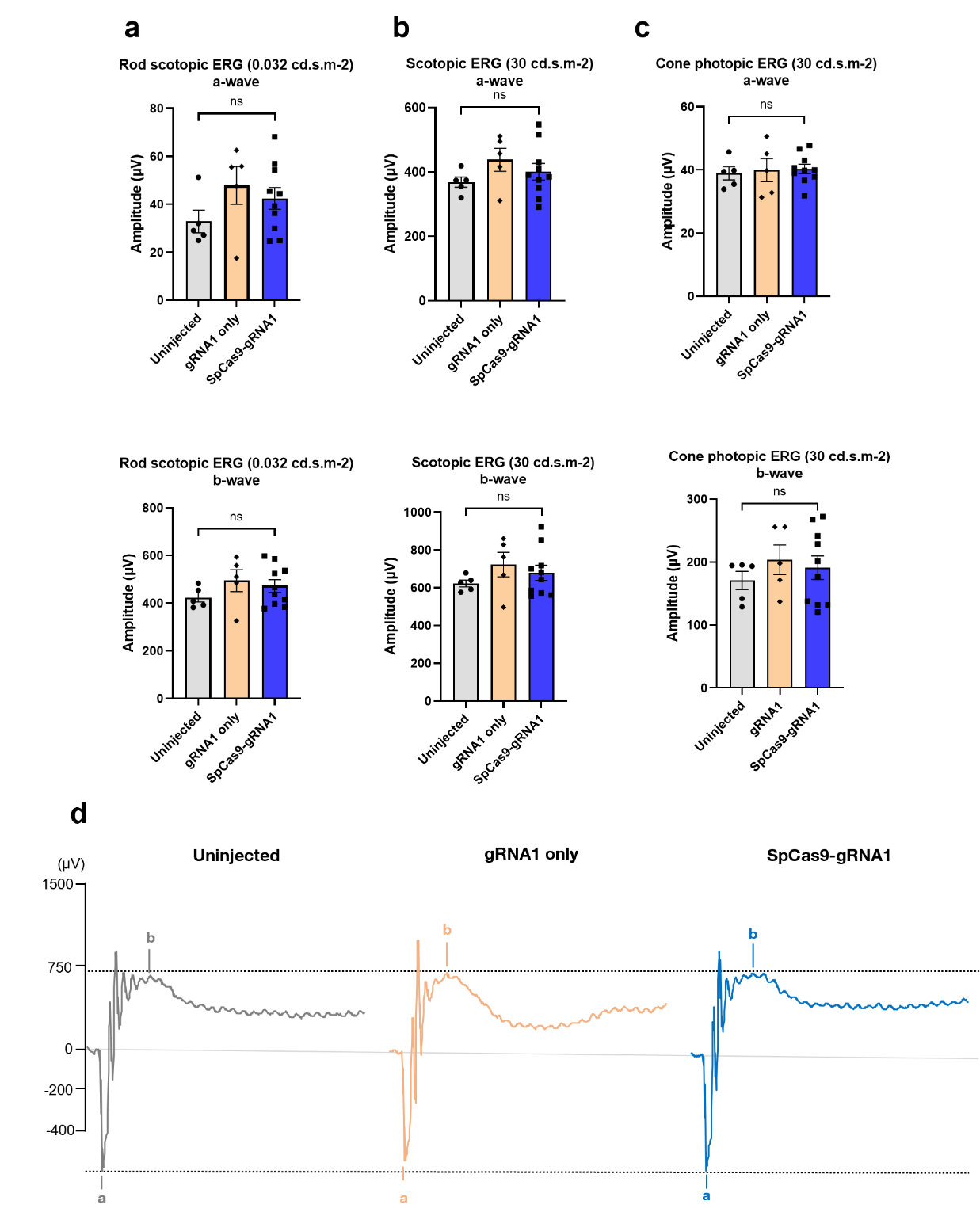


**Supplementary figure 5. 5’UTR genomic modification showed harmlessness to the visual function of wild-type mice. a.** a- and b-wave rod scotopic ERG response of wild-type mice that were untreated (n=10) or treated with gRNA1 only (n=5) or SpCas9-gRNA1 (n=5). light intensity 0.032 cd.s.m^-2^. **b.** a- and b-wave scotopic ERG response. light intensity 30 cd.s.m^-2^. **c.** a- and b-wave photopic ERG response. light intensity 30 cd.s.m^-2^. **d.** Representative electroretinography traces of untreated eye, eye treated with gRNA1 only and SpCas9-gRNA1. Data were presented as mean ± s.e.m, one-way ANOVA with Tukey post-hoc test (**a-c**).


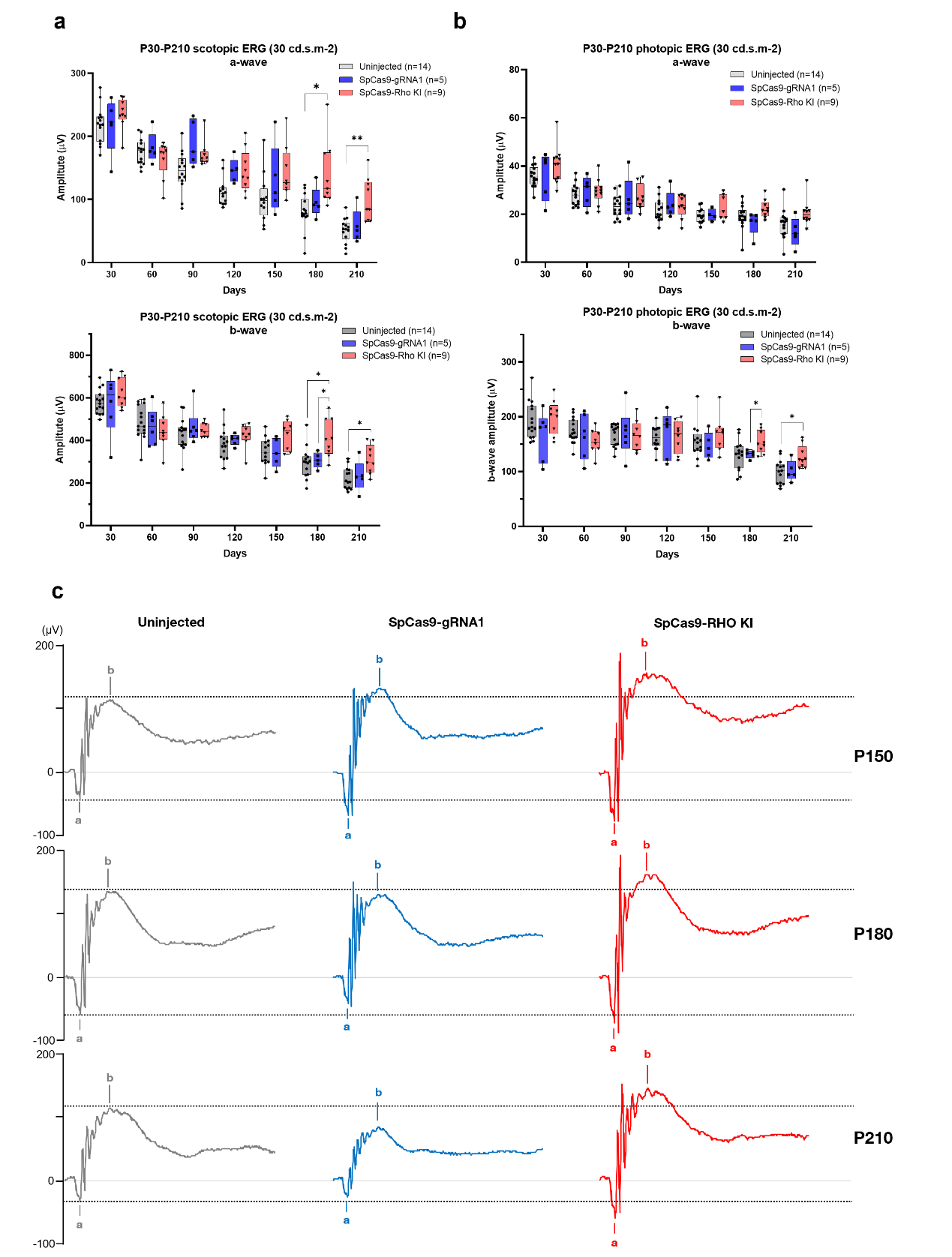


**Supplementary figure 6. 5’UTR *Rho* integration mediated by AAV-SpCas9 preserved visual function of *Rho^P23H/wt^* mice. a.** P30-P210 a- and b-wave amplitudes of scotopic ERG responses of control and treated *Rho*^P23H/wt^ eyes under light intensity 30 cd.s.m^-2^. **b.** P30-P210 a- and b-wave cone photopic ERG responses of control and treated *Rho^P23H/wt^* eyes under light intensity 30 cd.s.m^-2^. **c.** Representative P150-P210 electroretinography traces of untreated eye (left), eye treated with SpCas9-gRNA1 (middle) and eye treated with SpCas9-*Rho* KI (right). Data are presented as mean ± s.e.m. **P*<0.05; ***P*<0.01; ****P*<0.001, two-way ANOVA with Tukey post-hoc test (**a-b**).


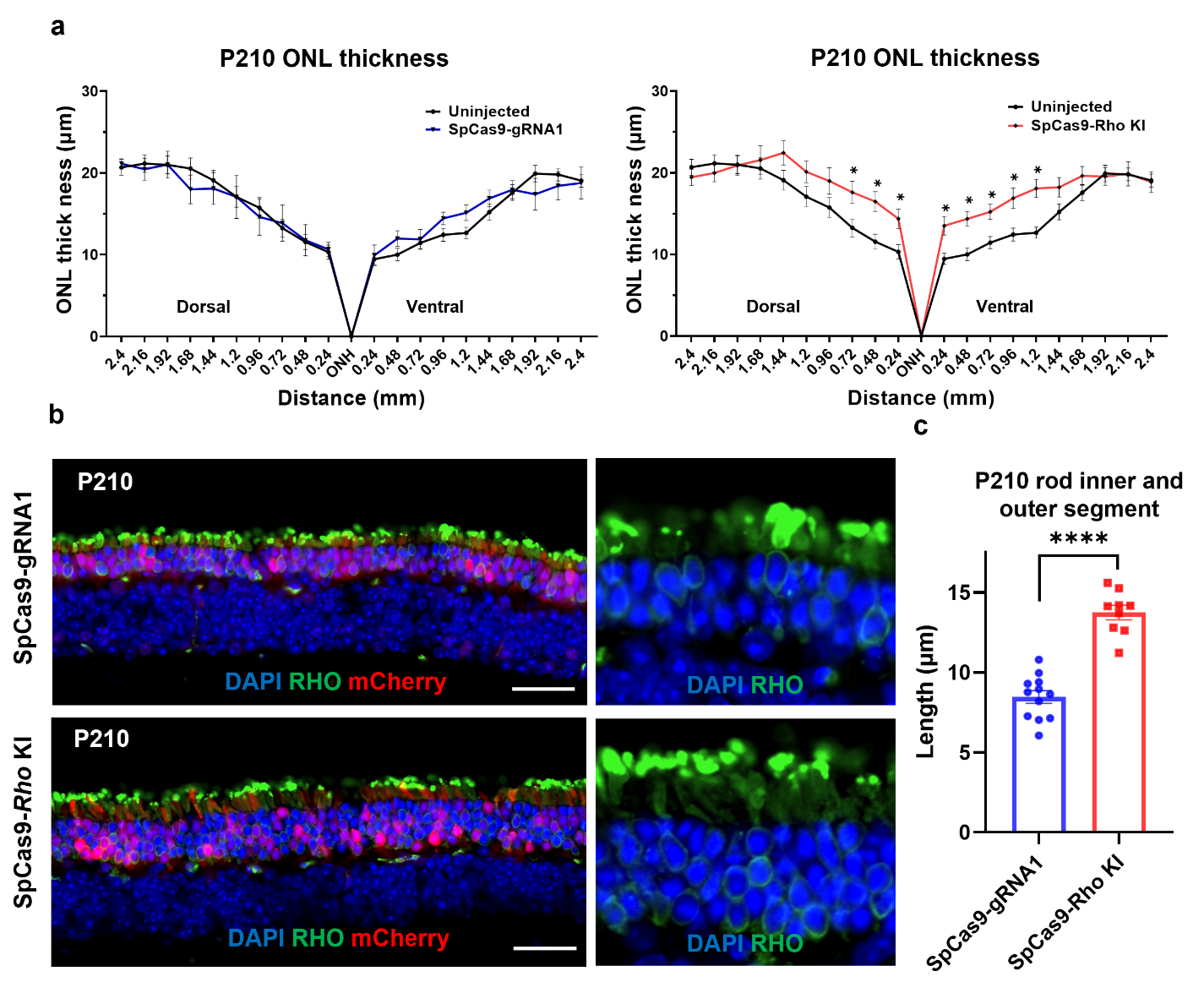


**Supplementary figure 7.** **5’UTR *Rho* integration mediated by AAV-SpCas9 slowed the degeneration of photoreceptors of *Rho^P23H/wt^* mice. a.** ONL thickness quantification of P210 *Rho*^P23H/wt^ retinas at different distances from optic nerve head (ONH). Untreated group (*n*=14); SpCas9-gRNA1 (*n*=5); SpCas9-*Rho* KI (*n*=9). **b.** Zoom-in images of RHO staining of *Rho*^P23H/wt^ mice at the end point P210. Scale bar, 50 μm. **c.** P210 rod inner and outer segment length comparison between SpCas9-gRNA1 and SpCas9-*Rho* KI. Data are presented as mean ± s.e.m. **P*<0.05, *****P*<0.0001, unpaired two-tailed Student t-test (**c**) two-way ANOVA with Tukey post-hoc test (**a**).


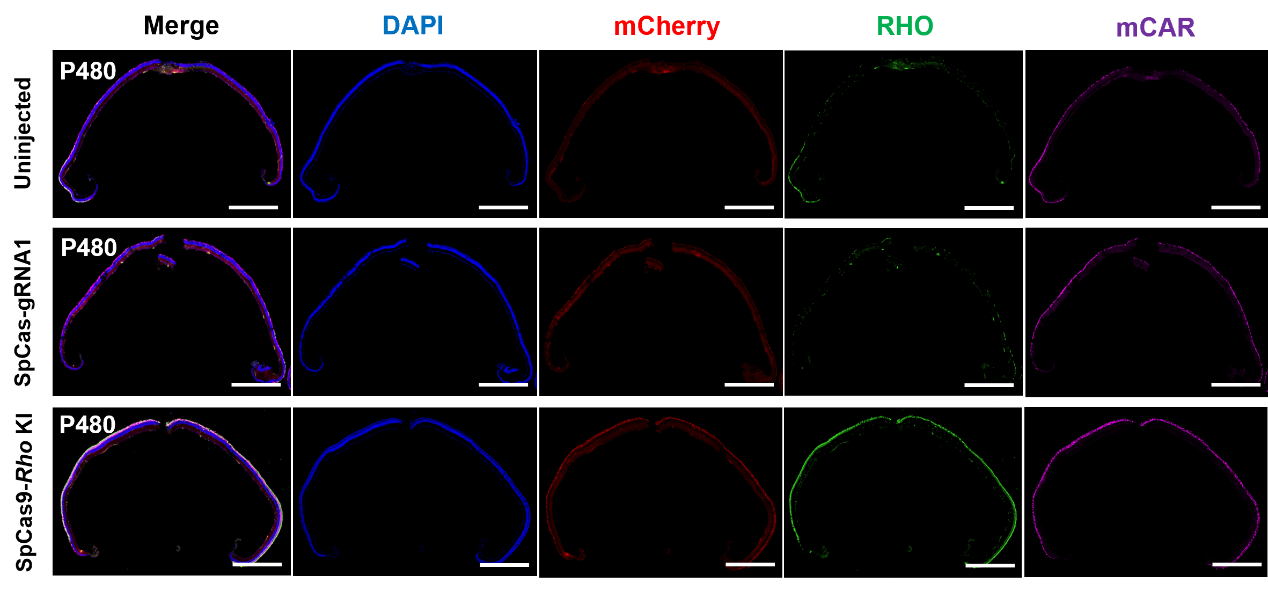


**Supplementary figure 8. SpCas9-*Rho* KI revealed a long-term protective effect for the photoreceptors of *Rho*^P23H/wt^ mice with chronic retinal degeneration.** Representative retinal immunostaining sections of P480 *Rho*^P23H/wt^ mice. Almost all rod and cone photoreceptors were apoptotic in untreated retina and retina treated with SpCas9-gRNA1 at the late stage of retinal degeneration. However, the photoreceptors of SpCas9-*Rho* KI were well preserved. Sections were immunostained with mCAR (cone marker) and RHO (rod marker) antibodies. Scale bar, 500 μm.


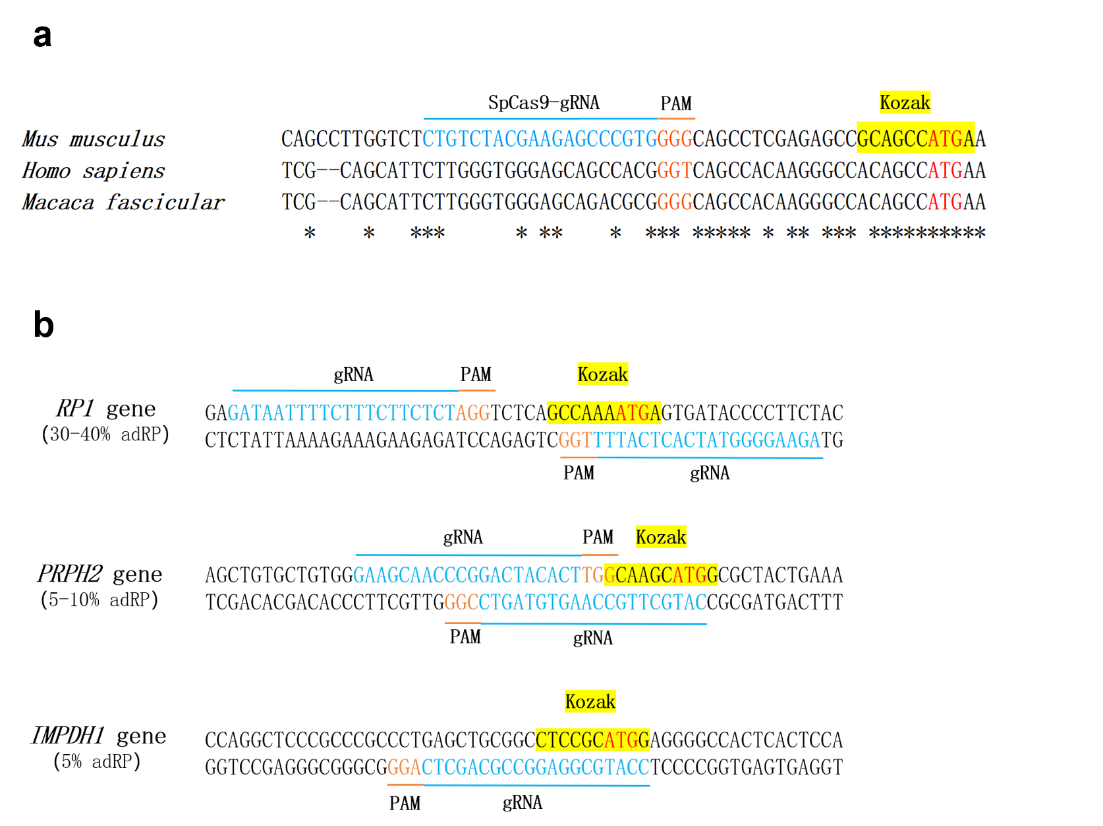


**Supplementary figure 9. PAM sites in the 5’UTR possess high cross-species conservation and easy accessibility. a.** *Rho* genomic DNA alignment between *Mus musculus*, *Macaca fascicular* and *Homo sapiens*. **b.** Potential SpCas9 target sites in 5’UTR of other human adRP associated genes. Sequence in blue, SpCas9-gRNA target regions. Asterisk, the same nucleobase. Sequence in orange, PAM site. Region highlighted by yellow, Kozak sequence.

Reference

1 Lem, J. *et al.* Morphological, physiological, and biochemical changes in rhodopsin knockout mice. *Proc Natl Acad Sci U S A*. **96**, 736-741, (1999).

2 Sakami, S. *et al.* Probing mechanisms of photoreceptor degeneration in a new mouse model of the common form of autosomal dominant retinitis pigmentosa due to P23H opsin mutations. *J Biol Chem*. **286**, 10551-10567, (2011).

3 Mai, S. *et al.* Postnatal eye size in mice is controlled by SREBP2-mediated transcriptional repression of Lrp2 and Bmp2. *Development*. **149**, (2022).

4 Khani, S. C. *et al.* AAV-mediated expression targeting of rod and cone photoreceptors with a human rhodopsin kinase promoter. *Invest Ophthalmol Vis Sci*. **48**, 3954-3961, (2007).

5 Grieger, J. C., Choi, V. W. & Samulski, R. J. Production and characterization of adeno-associated viral vectors. *Nat Protoc*. **1**, 1412-1428, (2006).

6 Clement, K. *et al.* CRISPResso2 provides accurate and rapid genome editing sequence analysis. *Nat Biotechnol*. **37**, 224-226, (2019).

7 Goto, Y. An electrode to record the mouse cornea electroretinogram. *Doc Ophthalmol*. **91**, 147-154, (1995).

8 Kinoshita, J. & Peachey, N. S. Noninvasive Electroretinographic Procedures for the Study of the Mouse Retina. *Curr Protoc Mouse Biol*. **8**, 1-16, (2018).
